# Big mice die young but large animals live longer

**DOI:** 10.18632/aging.100551

**Published:** 2013-04-20

**Authors:** Mikhail V. Blagosklonny

**Affiliations:** Department of Cell Stress Biology, Roswell Park Cancer Institute, BLSC, L3-312, Elm and Carlton Streets, Buffalo, NY, 14263, USA

**Keywords:** MTOR, rapamycin, aging, geroconversion, gerosuppression

## Abstract

It has been known for millennia that large animals live longer, inspiring numerous theories of aging. For example, elephants and humans live longer than mice, which in turn live longer than worms and flies. The correlation is not perfect, with many explainable exceptions, but it is still obvious. In contrast, within each species (e.g., mice and some other mammals) small body size is associated with longevity and slow aging. The concept that aging (and age-related diseases) is an aimless continuation of developmental growth, a hyperfunction driven by the same nutrient-sensing and growth-promoting pathways such as MTOR, may explain this longstanding paradox.

## INTRODUCTION

The excellent study by Miller et al “Big mice die young: early life body weight predicts longevity in genetically heterogeneous mice” [1] has set in stone the paradigm that large mice live shorter [2-9]. Weight in 2-month-old mice is a significant predictor of life span [1]. The rule is common in mammals, within (but not between) species, and even including humans in some special studies [10-16] (note: we will also discuss opposite tendency favoring taller people to live longer).

Yet, it has been known for millennia and is absolutely obvious that larger animals of different species live longer. Elephants live longer than mice and 75 ton bowhead whales may live up to 150-200 years, which is at least 3000 times longer than the lifespan of small *C. elegans*. So how do two rules co-exist? Based on a new view on aging, named for brevity “hyperfunction theory” [17-19], we can explain why and how the two phenomena coexist. Various applications and aspects of hyperfunction theory have been already extensively reviewed [19-35] (with references on other articles within), so here only a brief summary is needed in order to answer a particular question: “Why big mice die young but large animals live longer”.

Mechanistic (formally, mammalian) Target of Rapamycin (MTOR) is activated by nutrients (glucose, amino acids, fatty acids), oxygen, hormones (such as insulin), growth factors and cytokines and, in turn, stimulates growth and metabolism and is involved in pathological conditions such as diseases of aging [20, 38-53].

MTOR mechanistically links cellular mass growth and senescence, whereas aging is a continuation of growth [26, 54]. When actual cellular growth becomes impossible (post-mitotic and arrested cells), MTOR drives cellular aging/senescence [55-61], a process named geroconversion [62]. Importantly, MTOR stimulates cellular functions such as secretion and lipogenesis [63-65]. Senescent cells are hyperfunctional, leading to age-related diseases and conditions, an increasing the probability of death (organism aging) [34, 62]. This topic is beyond the scope of this article, it was discussed before [34] and cannot be discuss here in detail.

**Figure 1:**
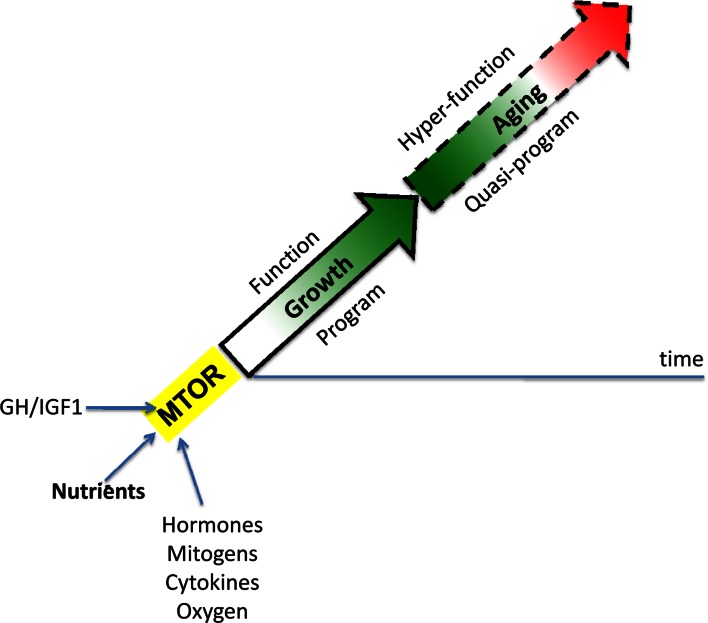
MTOR-driven quasi-programmed aging (hyperfunction model) Aging is a continuation of developmental growth driven by growth-promoting pathways such as MTOR. Green: robustness. Red: hyperfunction-driven diseases leading to death. See Ref. 19.

When the developmental program of an organism is completed, full MTOR activity is not needed. Then, instead of actual growth, MTOR drives aging and age-related diseases [19, 20]. Thus, aging is an aimless continuation of developmental programs (Figure 1), driven by the same “MoTOR” in the same direction (at first) and may be at almost the same speed [27]. A quasi-program for aging is not a program (it has no purpose) but a blindly-running program of developmental growth that has been already completed but not switched off [31]. At least initially, the MTOR-driven quasi-program causes no visible harm to the organism. In humans, adulthood may seem very healthy indeed, despite subclinical changes of homeostasis [19]. Overt diseases and organ damages arise much later in life. But natural selection is not at play at such ages, because rare animals survive until deep aging in the wild. Exceptions will be discussed here.

### Why do large animals live longer?

**Figure 2:**
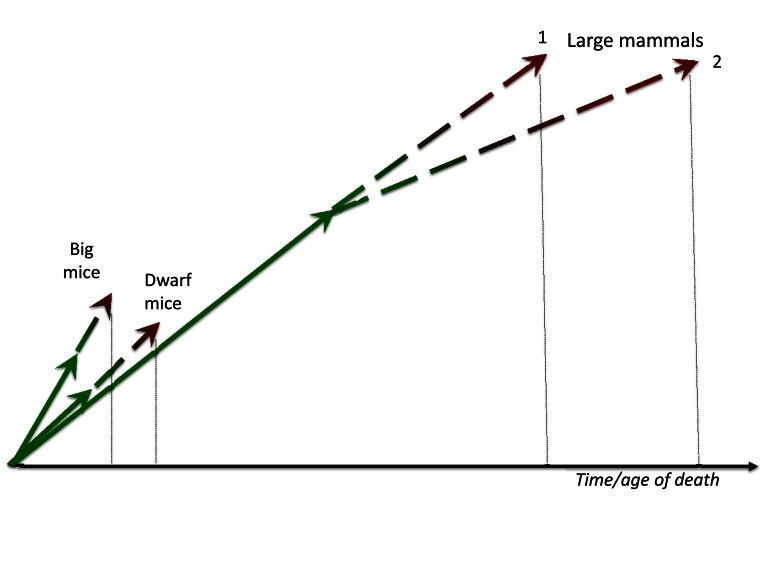
Big mice and large mammals Big mice grow faster than normal and especially dwarf mice. Fast growth is translated in fast aging. In contrast, large mammals develop for a prolonged period of time and aging (a continuation of developmental growth) is slow too. Given that very large animals may have very low extrinsic death rate and therefore may die from aging, natural selection may, in theory, favor deceleration of aging (curve 2).

Larger animals live in more protected environments with less accidental death from extrinsic causes, so natural selection favors slow aging. For example, elephants or whales (unless hunted by men) may die from “old age”: so natural selection has been at work to further increase their life span. Large size by itself is protective from predators. Large, complex organisms with sophisticated behaviors require prolonged periods of development, so large animals develop slower. Then aging, a continuation of developmental growth, is also slow (Figure 2). Second, due to low extrinsic death rate, natural selection may favor slow aging in large animal types, and the most natural way to do so is to repress growth-promoting/gerogenic pathways such as MTOR. This will automatically decelerate developmental growth: the most certain way to slow aging is to slow the developmental growth. In other words, slow development turns into the slow quasi-program of aging and age-related diseases. Taking these two reasons together, there is a positive feedback loop between slow development and low incidental death rate, ensuring that large animals are long-lived. **In brief, species with large body size and low accidental death rate have undergone (or even undergo now) selection for longevity. Because aging is a quasi-progammed hyperfuntcion and continuation of growth, slow aging can be a result of slow development in large animals**.

### Why the correlation is not perfect

Yet low accidental death rate can be dissociated from body size. Then, even smaller animals, which live in protected environments in the wild, live longer than equally-sized animals. Examples include bats and naked mole-rats, compared with rats. In such animals, natural selection favors slow aging and aging-tolerance (the term discussed in [27, 33]). Hypothetically, in durable safe environments with low accidental death rates, natural selection may favor a “decelerator” of MTOR, slowing down aging after development. Another way is to increase aging-tolerance, despite the same rate of aging. For example, changes in the developmental program that increases the number of collateral arteries in vital organs such as the heart (aging-tolerance) can extend lifespan despite the same rate of atherosclerosis (aging).

### Why do big mice age faster?

Big mice grow faster than slow-growing mice. They are bigger than slow-growing counterpart at the same age [1]. This is an advantage earlier in life in any environment. (Note: one may argue that this may be disadvantage in the wild because large mice need to eat more to stay alive. However, a cause-effect relationship may be opposite: it is food, which activates MTOR and growth, that makes mice bigger at the time of plenty). But if they are larger because nutrient- and growth factor-sensing pathways like MTOR are over-active, then such fast-growing mice should age faster too (Figure 2).

Calorie restriction decreases size, prolongs development, delays reproduction and, on the other hand, delays aging, age-related diseases, loss of reproduction [28, 66]. In contrast, by stimulating MTOR, overeating causes the opposite effects. While nutrients, insulin, growth factors all activate the MTOR pathway, they accelerate gerogenic conversion and hyperfunctional aging.

Similarly, mice overproducing growth hormone and IGF are bigger and live shorter lives [6-70]. In contrast, small (slow-growing) mice which have defects at different points of the growth pathway (such as RasGrf1−/−, growth hormone-deficient, GH receptor-deficient, IRS1−/−, S6K1−/−) live longer [5, 8, 71-78]. For all these examples, short life span is associated with increased MTOR activity, while long-lived strains have a decreased MTOR activity [36]. This was discussed in detail [36]. Whether long-lived strains are insulin sensitive (see Figure 2 in Ref. [36] or insulin-resistant (Figure 3 in Ref. [36]) is co-incidental. Thus, insulin resistance may result from MTOR overactivation (bad condition). Other types of insulin/IGF1 resistance may result in deactivation of MTOR (benevolent condition) (see figures in [36]).

The activity of MTOR, in theory, determines both body size and lifespan, as discussed in detail [19-35] (with references on other articles within).

Noteworthy, fibroblasts from long-lived mutant mice exhibit lower MTOR activity [79]. In contrast, in the muscle of long-lived Ames dwarf mice, the PI3K/Akt/MTOR pathway is deactivated compared with their normal size siblings at the same age of 2 months [80].

### In sum, to achieve prematurely big body weight, a mouse needs hyperactive growth-promoting pathways (genetic mutation, overfeeding), which later drives accelerated aging. This happens because two processes are continuation of one another and because growth-promoting pathways such as MTOR are involved in both growth and aging (Figure 2)

Importantly, slow-aging mice were developed in the laboratory. Small size and late reproduction and infertility are disadvantages in the wild with high risk of accidental death. Yet slow-growing, slow-aging GH- and GH receptor-deficient mice are an excellent model to illustrate that aging is a continuation of developmental growth and is driven by the same growth-promoting signals. What if this gerogenic force could be diminished to decelerate aging after the completion of development. In an outstanding study by the Miller group [81], long-lived Snell dwarf mice received 11 weeks of GH that increased their weight, although they remained much smaller than controls. The treatment also restored fertility to male dwarf mice and did not diminish life span [81]. I suggest that GH induces IGF-I and thus activates the MTOR pathway [82], which drives growth and then aging [26].

IGF levels correlates with body size and short longevity in mice [83-85]. In the Miller study, GH had been discontinued before mice reached full size and therefore only developmental growth was affected and promoted. Aging remained slow. The quasi-program of aging was not affected. (Another study, however, demonstrated that seemingly similar treatment with GH affected the speed of aging [86]. The difference may depend on dosage, duration and age of treatment [86]. As noted by Panici *et al* [86], the dosages were effectively declining in the Miller study [81]. Also, the age of nutritional intervention may switch the mouse to a slow aging trajectory [87].

And vice versa, deceleration of a quasi-program can be achieved in normal mice by administration of rapamycin, even when developmental growth program has been completed [88-90].

### Males age faster than females

In many species, males are bigger than females. Could that be a special case of the rule that “big mice age fast.” Males must be strong and robust because of the competition for mates, fights and dangerous behavior. In the wild, an accidental death rate is very high for young males, so there is no need to age slower, especially given that fast aging is “a continuation” of the program of robustness and growth. Natural selection does not need to turn off the “MoTOR of aging” (MTOR). Especially because, as discussed previously [91, 92], MTOR brings about robustness earlier in life. Besides increasing strength and muscle size, testosterone stimulates MTOR. And rapamycin decreases both spermatogenesis and testosterone, although completely reversibly [93]. So low MTOR is a disadvantage for young males, where high MTOR activity is an advantage, providing hypertrophy and increased of functions. But hypertrophic functions become later hyperfunctions, causing loss of homeostasis, diseases and organ damage. In sum, males age faster and develop age-related diseases earlier than females. It was recently shown that young male mice have increased MTOR activity in the heart and the liver, and this activity correlates with body weight [94]. This supports the hypotheses explaining why males live shorter [94].

### Emergence of slow-aging individuals in extremely protected environment

Several studies demonstrated that people with low body weight live longer. This could be explained by the low activity of the GH/IGF1/MTOR pathway, consistent with the “within species” rule. On the other hand, human life span is constantly increasing but people become taller. One hypothetical explanation is that (in the past) long-developing individuals died young from infections, starvation and accidents [95]. Now slow-developing and therefore slow-aging individuals survive until aging: the average lifespan is increasing. In principle, as speculated, their phenotype can be associated with “weak” MTOR and prolonged development [95]. In analogy, *C. elegans* lacking PI3K, an upstream component of the MTOR pathway, have prolonged developmental times and (under very protective environments) they mature into extremely long-lived adults (10-fold extension of both median and maximum adult lifespan) [96]. In humans, delayed puberty (slow development) is associated with exceptional longevity [97].

### Instead of conclusion

Fast versus slow aging may depend on whether the organism “grows fast” or “develops longer”: first case should be associated with high MTOR. Exceptions may be numerous. Small size is not always related to the GH/IGF/MTOR pathway but instead may be caused by defects that shorten life span. But understanding of each exception will further illuminate the rules [98, 99]. On a wider scale (from worm to whale), large animals live longer because aging is quasi-programmed. In contrast, “big” mice live shorter because they grow faster than dwarf mice and growth is driven by the same pathways that drive aging. Fast-growing mice are expected to have over-activation of growth-promoting pathways (either by excessive calorie consumption or due to genetic mutations), which drive aging and age-related diseases later. Cellular hyperfunction is the key feature of aging cell, leading to organismal death [17-19] Yet, there are also two other crucial aspects of hyperfunction theory: (a) aging as a quasi-program of developmental growth and (b) both processes are driven by the same growth-promoting-signaling pathways including MTOR.
